# Cell-Free DNA Methylation of Selected Genes Allows for Early Detection of the Major Cancers in Women

**DOI:** 10.3390/cancers10100357

**Published:** 2018-09-26

**Authors:** Sandra P. Nunes, Catarina Moreira-Barbosa, Sofia Salta, Susana Palma de Sousa, Inês Pousa, Júlio Oliveira, Marta Soares, Licínio Rego, Teresa Dias, Jéssica Rodrigues, Luís Antunes, Rui Henrique, Carmen Jerónimo

**Affiliations:** 1 Cancer Biology & Epigenetics Group—Research Center, Portuguese Oncology Institute of Porto (CI-IPOP), 4200-072 Porto, Portugal; sandra22nunes@hotmail.com (S.P.N.); catarina.moreira.barbosa@gmail.com (C.M.-B.); sofia.salta@ipoporto.min-saude.pt (S.S.); rmhenrique@icbas.up.pt (R.H.); 2 Master in Oncology, Institute of Biomedical Sciences Abel Salazar, University of Porto (ICBAS-UP), 4050-313 Porto, Portugal; 3 Breast Cancer Clinic and Department of Medical Oncology, Portuguese Oncology Institute of Porto, 4200-072 Porto, Portugal; susana.sousa@ipoporto.min-saude.pt; 4 Lung Cancer Clinic and Department of Medical Oncology, Portuguese Oncology Institute of Porto, 4200-072 Porto, Portugal; ines.ruiz@ipoporto.min-saude.pt (I.P.); julio.oliveira@ipoporto.min-saude.pt (J.O.); martasoares@ipoporto.min-saude.pt (M.S.); 5 Digestive Tract Pathology Clinic and Surgical Oncology, Portuguese Oncology Institute of Porto, 4200-072 Porto, Portugal; licinio@ipoporto.min-saude.pt (L.R.); teresa.dias@ipoporto.min-saude.pt (T.D.); 6 Department of Epidemiology, Portuguese Oncology Institute of Porto, 4200-072 Porto, Portugal; jessica.rocha.rodrigues@ipoporto.min-saude.pt (J.R.); luis.antunes@ipoporto.min-saude.pt (L.A.); 7 Department of Pathology, Portuguese Oncology Institute of Porto, 4200-072 Porto, Portugal; 8 Department of Pathology and Molecular Immunology, Institute of Biomedical Sciences Abel Salazar, University of Porto (ICBAS-UP), 4050-313 Porto, Portugal

**Keywords:** breast cancer, colorectal cancer, lung cancer, DNA methylation, epigenetic biomarker, cell-free DNA, liquid biopsy, detection

## Abstract

Background: Breast (BrC), colorectal (CRC) and lung (LC) cancers are the three most common and deadly cancers in women. Cancer screening entails an increase in early stage disease detection but is hampered by high false-positive rates and overdiagnosis/overtreatment. Aberrant DNA methylation occurs early in cancer and may be detected in circulating cell-free DNA (ccfDNA), constituting a valuable biomarker and enabling non-invasive testing for cancer detection. We aimed to develop a ccfDNA methylation-based test for simultaneous detection of BrC, CRC and LC. Methods: CcfDNA from BrC, CRC and LC patients and asymptomatic controls were extracted from plasma, sodium-bisulfite modified and whole-genome amplified. *APC*, *FOXA1*, *MGMT*, *RARβ2*, *RASSF1A*, *SCGB3A1*, *SEPT9*, *SHOX2* and *SOX17* promoter methylation levels were determined by multiplex quantitative methylation-specific PCR. Associations between methylation and standard clinicopathological parameters were assessed. Biomarkers’ diagnostic performance was also evaluated. Results: A “PanCancer” panel (*APC*, *FOXA1*, *RASSF1A*) detected the three major cancers with 72% sensitivity and 74% specificity, whereas a “CancerType” panel (*SCGB3A1*, *SEPT9* and *SOX17*) indicated the most likely cancer topography, with over 80% specificity, although with limited sensitivity. Conclusions: CcfDNA’s methylation assessment allows for simultaneous screening of BrC, CRC and LC, complementing current modalities, perfecting cancer suspects’ triage, increasing compliance and cost-effectiveness.

## 1. Introduction

Breast (BrC), colorectal (CRC) and lung (LC) cancers are the most incident and lethal malignancies affecting women from developed countries [1]. Over the last years, BrC incidence increased, fueled by mammography-based screening [1], which endows about 85% sensitivity and 90% specificity [2]. Nonetheless, sensitivity is much lower in dense breasts (over 70% of the breast tumors are missed in dense breasts) [3] and, although mammography may reduce mortality by 28–45% [4], it entails overdiagnosis (an estimated 11% in screening programs) and consequent overtreatment [5]. About 95% of CRC patients might be cured through surgery if diagnosed early and several screening protocols coexist [6]. Colonoscopy-based screening is performed every ten years after the age of 50 years [7], displaying high sensitivity and the main advantage of removing lesions at the time of detection [7]. However, it is an invasive and costly procedure, requiring unpleasant bowel preparation, sedation, the risk of bowel perforation, bleeding, and low compliance [7]. Fecal occult blood (FOBT) and fecal immunochemical (FIT) testing for triage of patients requiring subsequent colonoscopy are less-invasive options which have been shown to reduce CRC-related-mortality [8]. Nevertheless, the high false-positive rate is a limitation, since bleeding might derive from non-neoplastic disorders such as hemorrhoidal or inflammatory bowel diseases [8]. LC has become a major cause of cancer-related death among women in more developed regions [1], since 75% of the cases are diagnosed at advanced stages [9]. Low-dose computed tomography (LD-CT) has been suggested for LC screening. The National Lung Screening Trial comparing the performance of LD-CT with chest x-ray for LC screening found a 20% decrease in LC-related mortality in the LD-CT group, although without additional confirmatory studies [10] and at the cost of 96.4% false positive rate [11]. Thus, although current screening strategies for BrC, CRC and LC are beneficial, they may impact negatively on health systems management and women’s quality of life. Hence, the development of better pre-screening methods, which might prevent selection of invasive/costly screening tests, avoiding overdiagnosis/overtreatment and unnecessary procedures is necessary.

Aberrant promoter methylation of cancer-related genes is common at the earliest steps of carcinogenesis, thus constituting a source for promising cancer detection biomarkers [12]. This is a stable genomic alteration which might be detected in serum/plasma circulating cell-free DNA (ccfDNA) [13], that might even portray tumor heterogeneity better than tissue biopsies [14]. Several methylated genes have been proposed as tumor biomarkers for BrC, CRC or LC detection, including *APC*, *RARβ2* and *RASSF1A* [15,16,17,18]. Although *SEPT9* and *PTGER4*/*SHOX2* methylation-based non- or minimally invasive tests are already commercially available for CRC and LC detection, respectively [19,20], they have limited sensitivity. Furthermore, a recent study using methylation scores displayed 87% sensitivity for advanced cancer detection (BrC, CRC, non-small cell LC and melanoma) with 100% specificity, predicting also the cancer type in 76% of cases [21]. Thus, we aimed to develop a sensitive and specific methylation-based test enabling the simultaneous detection of BrC, CRC and LC in women using ccfDNA. For that purpose, promoter methylation levels of 9 genes (*APC*, *FOXA1*, *MGMT*, *RARβ2*, *RASSF1A*, *SCGB3A1*, *SEPT9*, *SHOX2* and *SOX17*), selected based on our previous experience [16,18] and extensive literature review [15,17,22,23,24,25,26,27,28,29], were assessed by multiplex quantitative specific PCR (qMSP) in ccfDNA extracted from plasma samples of female subjects.

## 2. Results

### 2.1. Clinical and Pathological Data

This study included 253 female patients with BrC (*n* = 108), CRC (*n* = 72) or LC (*n* = 73) and 103 female asymptomatic controls (AC). Detailed clinical and pathological characterization is provided in Table 1. Globally, the median age of cancer patients significantly differed from that of controls (*p* < 0.0001), and, thus, correlations between age and gene promoter methylation levels were assessed stratifying for ACs and cancer patients. Although *SOX17* promoter’s methylation levels correlated with controls’ age (R = 0.225, *p* = 0.009), this was not observed in cancer patients and no other significant correlations were disclosed.

### 2.2. Gene Promoter Methylation Levels in ccfDNA

**Figure 1:**
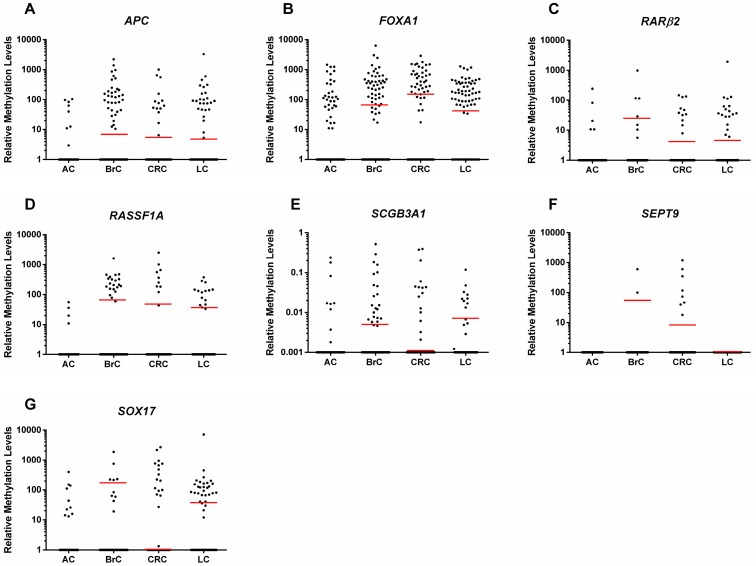
Scatter plot of the distribution of (**A**) *APC*, (**B**) *FOXA1*, (**C**) *RARβ2*, (**D**) *RASSF1A*, (**E**) *SCGB3A1*, (**F**) *SEPT9* and (**G**) *SOX17* relative methylation levels [(gene/β-Actin) × 1000] of breast cancer (BrC) (*n* = 108), colorectal cancer (CRC) (*n* = 72), lung cancer (LC) samples (*n* = 73) and asymptomatic controls (ACs) samples (*n* = 103). Red horizontal lines represent cut-off values.

*APC*, *FOXA1*, *RASSF1A* and *SCGB3A1* promoters depicted significantly higher methylation levels in BrC patients than in controls (*p* < 0.0001, *p* = 0.0063, *p* = 0.0003 and *p* = 0.0245, respectively) (Figure 1, Supplementary Table S1). Nonetheless, no significant differences were found for *MGMT*, *RARβ2*, *SHOX2*, *SEPT9* and *SOX17*.

In CRC patients, *APC*, *FOXA1*, *RARβ2*, *RASSF1A*, *SCGB3A1*, *SEPT9* and *SOX17* methylation levels were significantly higher than in controls (*p* = 0.005, *p* < 0.0001, *p* = 0.009, *p* = 0.012, *p* = 0.003, *p* = 0.001 and *p* = 0.007, respectively) (Figure 1, Supplementary Table S1), although no differences were apparent for *MGMT* and *SHOX2* methylation levels.

Concerning LC, significantly higher methylation levels compared to controls were disclosed for *APC*, *FOXA1*, *RARβ2*, *RASSF1A* and *SOX17* (*p* < 0.0001 for all genes), only (Figure 1, Supplementary Table S1).

### 2.3. Association Between Promoters’ Methylation Levels and Clinicopathological Features

**Figure 2:**
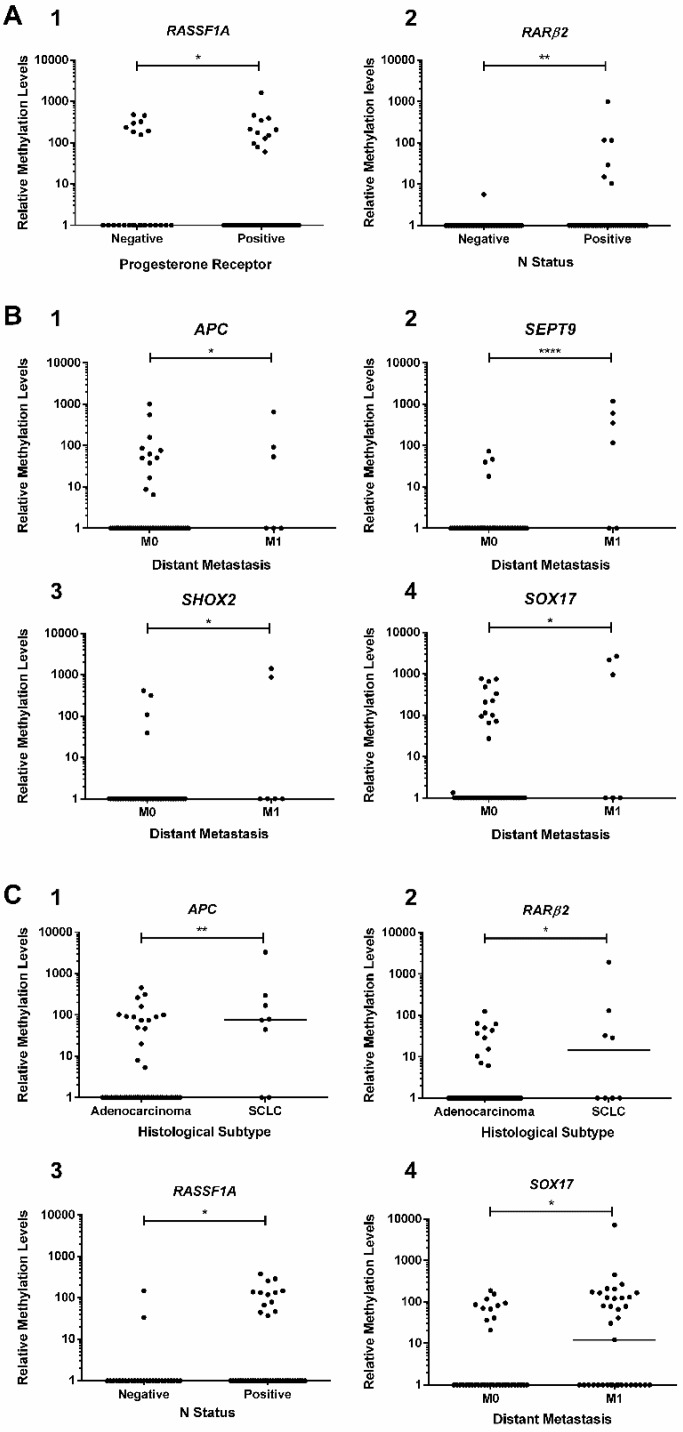
Scatter plot of (**A**) (1) *RASSF1A* promoter’s methylation levels in PR-positive or negative breast cancer (BrC) patients (Negative *n* = 24, Positive *n* = 94); (2) *RARβ2* promoter’s methylation levels between regional node (N) status in BrC patients (Negative *n* = 65, Positive *n* = 43), (**B**) (1) *APC*; (2) *SEPT9*; (3) *SHOX2* and (4) *SOX17* promoter’s methylation levels between metastatic (M1) and non-metastatic colorectal cancer (CRC) patients (M0) (M0 *n* = 66, M1 *n* = 6), and (**C**) (1) *APC* and (2) *RARβ2* promoters methylation levels for histological subtype [Adenocarcinoma *n* = 56, Small-cell Lung Cancer (SCLC) *n* = 8], (3) *RASSF1A* promoter’s methylation levels for regional node (N) status in lung cancer (LC) patients (Negative = 27, Positive = 45) and (4) *SOX17* promoter’s methylation levels between metastatic (M1) and non-metastatic LC patients (M0) (M0 *n* = 36, M1 *n* = 37). Mann Whitney U, n.s. *p* > 0.05, * *p* < 0.05, ** *p* < 0.01, **** *p* < 0.0001. Black horizontal line represents the methylation levels’ median.

Methylation levels of tested gene promoters associated with several clinicopathological features. Specifically, in BrC patients, *RASSF1A* methylation levels significantly differed between progesterone receptor (PR)+ and PR− tumors (*p* = 0.031) (Figure 2(A1)), whereas *RARβ2* promoter methylation levels were higher in node-positive than in node-negative BrC patients (*p* = 0.009) (Figure 2(A2)). Moreover, in CRC patients, *SEPT9* promoter methylation levels were significantly higher in patients with stage IV or distant metastatic disease (M1) (*p* < 0.01, in all comparisons) (Figure 2(B2)). Similar results were depicted for *APC*, *SHOX2* and *SOX17* promoter methylation in metastatic vs. non-metastatic CRC patients (*p* = 0.0276, *p* = 0.0107 and *p* = 0.0242, respectively), although no differences were found for stage (Figure 2B). Concerning LC, significantly higher *APC* and *RARβ2* promoter methylation levels were apparent in small-cell lung cancer (SCLC) patients compared to those with adenocarcinoma (*p* = 0.005 and *p* = 0.035, respectively) (Figure 2(C1,C2)). Moreover, node-positive LC patients displayed higher *RASSF1A* methylation levels than node-negative LC patients (*p* = 0.018, Figure 2(C3)), whereas higher *SOX17* promoter methylation was observed in patients with systemic metastization (*p* = 0.029) (Figure 2(C4)).

### 2.4. Biomarker Performance in ccfDNA

**Figure 3:**
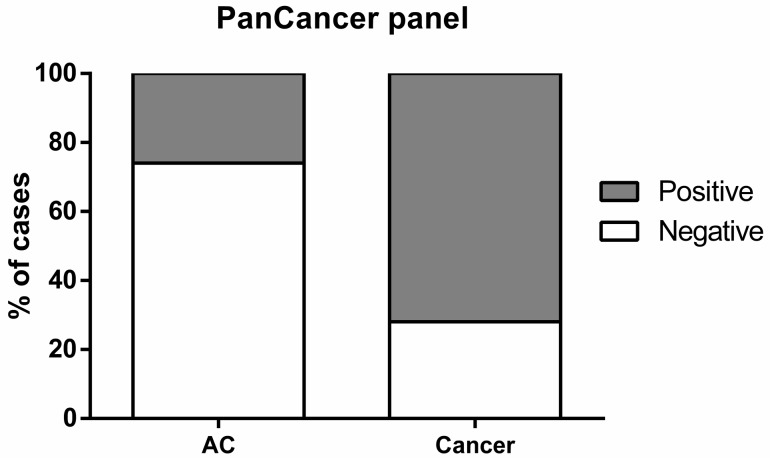
Percentage of cases identified by “PanCancer” panel in cancer samples (Positive 72%, Negative 28%) and asymptomatic controls (ACs) (Positive 26%, Negative 74%).

Gene promoters disclosing significantly higher methylation levels in cancer patients vs. controls were selected for assessment of BrC, CRC or LC detection performance in ccfDNA. *APC*, *FOXA1* and *RASSF1A* individually depicted sensitivity over 20% and specificity greater than 70%, for all cancers. *FOXA1* displayed the highest sensitivity (39% for BrC, 50% for CRC and 73% for LC). Overall *RASSF1A* disclosed the highest specificity (over 98%) for all three cancer types and *SEPT9* displayed 100% specificity for CRC detection. *SCGB3A1* detected BrC and CRC with over 20% sensitivity, whereas *RARβ2* and *SOX17* displayed specificity higher than 90% for CRC and LC detection (Supplementary Tables S2–S4). Since *APC*, *FOXA1* and *RASSF1A* were biomarkers common to BrC, CRC and LC, they were further tested as gene panel for cancer detection (designated “PanCancer”), whereas *RARβ2*, *SCGB3A1*, *SEPT9* and *SOX17* were considered a gene panel for discrimination of primary cancer localization (“CancerType” panel). In ccfDNA, the “PanCancer” panel correctly detected 183 out of 253 cancer cases, corresponding to 72.4% sensitivity, 73.5% specificity and 72.8% accuracy (Table 2, Figure 3).

**Figure 4:**
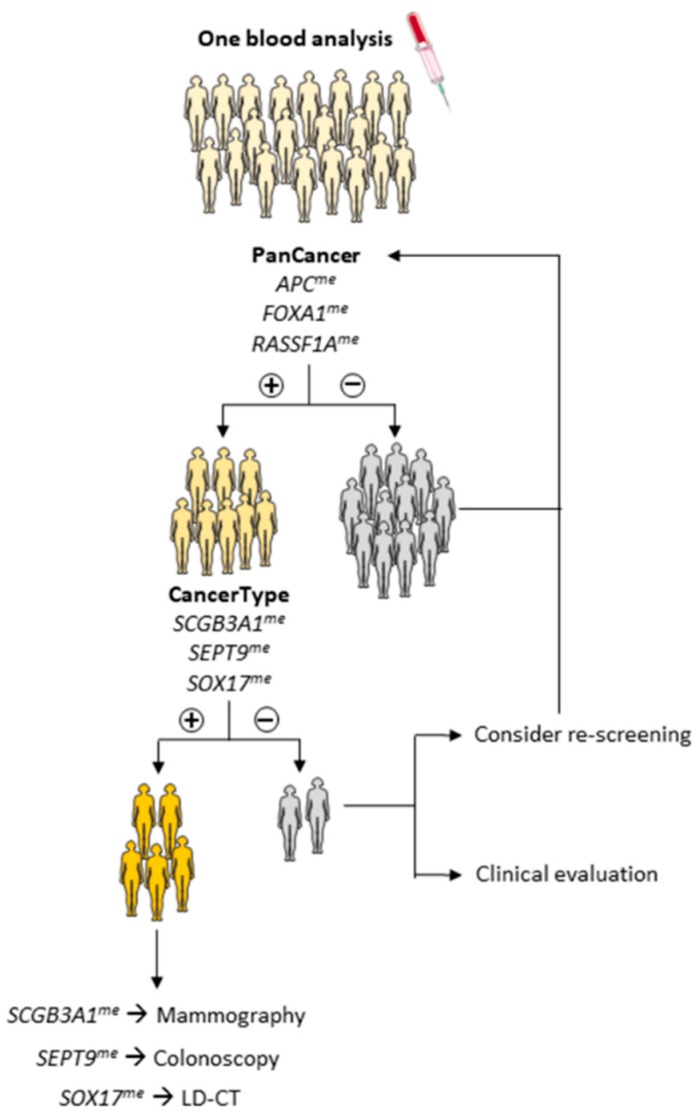
Schematic representation of a proposed algorithm for screening and management of breast, colorectal and lung cancers using the methylation tests. If “PanCancer” panel was positive, “CancerType” panel would be performed in order to determine the cancer type present. After “CancerType” panel, exams such as mammography, colonoscopy or low-dose computed tomography (LD-CT) would be executed to confirm the diagnosis. If “PanCancer” panel was negative, a re-screening would be proposed, whereas if “CancerType” panel was negative, a clinical evaluation or a re-screening would be considered as options.

Furthermore, “PanCancer” panel detected CRC stages 0, I and II with 78.4% sensitivity, 69.9% specificity, 48.3% PPV, 90.0% NPV and 72.1% accuracy and early LC with 85.7% sensitivity, 75.7% specificity, 41.9% PPV, 96.3% NPV and 77.4% accuracy. However only 37 CRC samples and 21 LC samples were used for this estimation, thus these results need further validation. Using the “CancerType” panel, three methylated genes might be used to indicate the most likely primary location of the tumor detected by the “PanCancer” panel (Table 3 and Table 4). *SCGB3A1* detected BrC with 80.0% specificity, whereas *SEPT9* methylation detected CRC with 98.9% specificity and *SOX17* detected LC with 85.1% specificity (Table 4). *RARβ2* was not further included in “CancerType” since it was not useful for discrimination between CRC and LC. The results of the “CancerType” panel could, then, be used to select the best strategy for identification of primary localization (mammography, colonoscopy or LD-CT) (Figure 4).

## 3. Discussion

BrC, CRC and LC are the most incident and lethal neoplasms among women in developed regions of the globe [1] and screening programs may decrease mortality through increased detection of early stage disease [4,6,7]. Mammography and colonoscopy are gold-standard for BrC and CRC screening, whereas LD-CT is recommended for high-risk smokers’ screening [4,30,31]. Notwithstanding, these screening tools have significant limitations, comprising risk of overdiagnosis/overtreatment, invasiveness and high cost, entailing low compliance, and suboptimal specificity, requiring further testing and increasing suspects’ anxiety [5,7]. Hence, low-invasive screening strategies, capable of better triaging cancer suspects for testing with highly specific methods are an important clinical challenge. Owing to the ubiquity and cancer-specificity of selected aberrant gene promoter methylation, enabling successful cancer detection in liquid biopsies [13], we assessed the feasibility of ccfDNA analysis using multiplex qMSP for simultaneous BrC, CRC and LC detection in women.

Candidate genes were selected based on an extensive and critical literature review [15,17,22,23,24,25,26,27,28,29], including our previously published results [16,18], and, globally, our findings are mostly in line with previous publications. For BrC, we confirmed *APC*, *FOXA1*, *RASSF1A* and *SCGB3A1* hypermethylation in ccfDNA, in accordance with published studies [15,23,24], whereas *RARβ2* methylation findings paralleled some previous studies [15,32], but not others, either in tissue [33], fine-needle washings [18] or serum [23,34]. Differences in methodology [34], population [23] and/or biological sample type [18,33] likely explain these dissimilarities. Furthermore, *SOX17* promoter has been reported as aberrantly methylated in ccfDNA and CTCs from BrC patients’ [35], albeit its BrC biomarker potential requires further investigation. As for CRC, the significantly higher *APC*, *FOXA1*, *RARβ2*, *RASSF1A*, *SCGB3A1*, *SEPT9* and *SOX17* methylation levels in cancer patients are in line with previous publications [16,27,36,37,38,39], although divergent results have been reported for *MGMT* [16,27]. Concerning LC, and except for *SHOX2,* our results are in accordance with previous studies [17,28,29,40,41]. To the best of our knowledge, this is the first study disclosing *FOXA1* methylation in CRC and LC patients’ ccfDNA.

Although several gene methylation panels have been proposed for specific cancer detection using ccfDNA [15,17,24,26], our main goal was to devise a gene panel enabling the simultaneous detection of the three most common cancers among women, thus potentially increasing the cost-effectiveness of a methylation-based screening test. Remarkably, similar sensitivity and specificity was disclosed by the “PanCancer” panel (*APC*, *FOXA1* and *RASSF1A*) compared to other gene methylation panels proposed for individual BrC, CRC and LC detection [15,17,24,26]. Compared to mammography, “PanCancer” discloses lower sensitivity and specificity [2], but it may be advantageous for triaging women for mammographic screening, eventually decreasing cumulative radiation exposure and costs, while increasing women’s compliance. It would be interesting to ascertain whether the molecular test might provide more accurate screening results than mammography in women with high breast density, for which mammography is mostly ineffective. Optimally performed colonoscopy detects CRC with 58–75% sensitivity, depending on the localization of the tumor [42], and allows for confirmatory tumor biopsy and polyp removal [30]. Nevertheless, it is a costly, invasive approach that requires prior preparation and sedation [30], whereas FOBT tests are non-invasive but have limited sensitivity and specificity [30]. The “PanCancer” panel disclosed similar detection performance to colonoscopy and superior to fecal occult blood tests, constituting a minimally-invasive test, amenable for screening. Finally, the “PanCancer” panel clearly outperformed LD-CT for LC detection and might be favorably used in a pre-screening context, to better identify high-risk suspects of harboring LC (Figure 4). Interestingly, the “PanCancer” panel detected stage I and II LC with a high sensitivity and specificity, and may, thus, constitute a novel option for LC early detection. Furthermore, it is likely that lesions that are difficult to diagnose by imaging techniques might be detectable using the “PanCancer” panel as Shan et al. have previously demonstrated that a methylation-based panel detected small breast tumors (<1 cm) with higher sensitivity than mammography [24].

Identifying the putative cancer primary localization following a positive “PanCancer” panel result constitutes the next challenge. Based on the individual performance of the remainder gene promoters tested, we proposed another panel (“CancerType”) which attempts to indicate the most likely topography of the primary tumor. To increase cost-effectiveness, this panel would only be performed in “PanCancer” positive cases, allowing cancer suspects to be directed for mammography, colonoscopy or LD-CT. Although “CancerType” genes individually display low sensitivity, the main goal of this panel is to discriminate among the three cancer types, requiring high specificity. Risk factors should be also considered (e.g., familial history of BrC or CRC, tobacco exposure) to improve the detection strategy. In cases in which no tumor is found, looking for the remaining possible localizations should be guided according to clinical evaluation. Repeat testing after a defined time could also be considered. It is difficult, however, to estimate how results of the “CancerType” panel would perform in a real setting as it assessment implies a carefully designed study with relatively long follow-up period.

Some interesting clinicopathological correlates with gene promoter methylation status were disclosed. The association of *RASSF1A* promoter methylation with PR status parallels previous reports [43] and higher *RARβ2* methylation in node-positive BrC is in line with previous findings in sentinel lymph node metastasis [44] and the correlation between primary BrC and lymph node metastasis tissues [45]. Furthermore, some of the tested candidate genes might also convey relevant prognostic information, as *APC*, *SEPT9*, *SHOX2* and *SOX17* methylation levels were increased in CRC patients with distant metastasis. Interestingly, a recent study disclosed higher *SEPT9* and *SHOX2* methylation levels in ccfDNA of CRC patients with distant metastasis and advanced stages [46]. Moreover, a correlation between *APC* methylation and more advanced CRC stage was previously established in CRC tissue analysis [47] and *APC* methylation was also found in CRC hepatic metastasis [48]. Another interesting finding was the higher *APC* and *RARβ2* methylation levels in patients with SCLC vs. lung adenocarcinoma. Recently, a microRNA-based test (miRview^®^) test was approved for discrimination among LC subtypes [49], based on analysis of pre-operative biopsies, which might be difficult to obtain. Thus, gene promoter methylation assessment in ccfDNA might prove advantageous in lung tumors with difficult access, since SCLC requires a specific treatment regimen and is associated with a worse prognosis [50]. Furthermore, an association between *RASSF1A* methylation and node-positive LC patients was found, which is in accordance with previous publications demonstrating higher *RASSF1A* methylation levels in more advanced tumor stage, associating with local recurrence and worse prognosis in LC patients [51,52]. Finally, *SOX17* promoter methylation levels are associated with distant metastasis, in agreement with previous studies using plasma samples from LC patients [40].

The main limitations of this study are the limited number of samples tested and the lack of long-term follow-up, which would be required to determine whether asymptomatic controls testing positive would subsequently develop BrC, CRC or LC. These limitations also preclude an accurate estimate of the use of the two gene panels in a “real world” scenario. Nevertheless, it should be emphasized that our proposal is innovative and might foster the development of more accurate and cost-effective tools for BrC, CRC and LC screening.

## 4. Materials and Methods

### 4.1. Patients and Samples Collection

Blood samples were collected from female patients (*n* = 253) with BrC (*n* = 108), CRC (*n* = 72) or LC (*n* = 73) at the time of diagnosis, prior to any treatment, and from female healthy donors [(asymptomatic controls (AC)] older than 45 years (*n* = 103), at the Portuguese Oncology Institute of Porto, Portugal. Plasma was separated from blood harvested in EDTA tubes by centrifuging at 2000 rpm for 10 min and immediately frozen at −80 °C. Relevant clinical and pathological data was retrieved from clinical charts and an anonymized database was constructed for analysis purposes (Table 1, Supplementary Table S5).

This study was approved by the institutional review board (Comissão de Ética para a Saúde–CES 120/2015) of Portuguese Oncology Institute of Porto, Portugal. All patients and healthy donors enrolled in this study provided written informed consent, in accordance with the Declaration of Helsinki ethical principles.

### 4.2. Ccf-DNA Extraction, Sodium-Bisulfite Modification and Whole Genome Amplification (WGA)

QIAamp MinElute ccfDNA (Qiagen, Hilden, Germany) was used for ccfDNA extraction from 2–3 mL of plasma, according to manufacturers’ instructions, subsequently eluted in 20 μL of sterile distilled water and stored at −20 °C until further use. All ccfDNA samples were bisulfite-modified using EZ DNA Methylation-Gold™ Kit (Zymo Research, Irvine, CA, USA) according to the manufacturer’s recommendations. Twenty µL of extracted ccfDNA and 1 μg of CpGenome™ Universal Methylated DNA (Merck Millipore, Burlington, MA, USA) were used for sodium-bisulfite modification. The bisulfite-converted ccfDNA was eluted in 10 µL of sterile distilled water and stored at −80 °C until further use. WGA of 10 µL sodium-bisulfite modified ccfDNA was carried out using the EpiTect Whole Bisulfitome Kit (Qiagen, Hilden, Germany) according to manufacturer’s recommendations [53,54]. Amplified DNA was diluted in 25 µL of sterile distilled water, in a final volume of 65 µL, and stored at −20 °C until further use. Extracted ccfDNA, amplified DNA and sodium-bisulfite converted DNA were quantified using Qubit 2 Fluorometer (Invitrogen, Carlsbad, CA, USA) following manufacturer’s instructions.

### 4.3. Multiplex qMSP

The nine genes (*APC*, *FOXA1*, *MGMT*, *RARβ2*, *RASSF1A*, *SCGB3A1*, *SEPT9*, *SHOX2* and *SOX17*) promoter methylation levels were assessed by multiplex qMSP, using amplified DNA as template [25,55]. Primers and probes specifically designed for the modified gene sequence plus the fluorochromes and quenchers used for each probe are listed in Supplementary Table S6. *β-Actin* was used as reference gene to normalize the DNA quantity of each sample [18]. 6 µL of WGA amplified DNA and Xpert Fast Probe (GRiSP, Porto, Portugal) were used in each multiplex qMSP reaction. Multiplex qMSP assays were carried out in 96-well plates in triplicate using a 7500 Sequence Detector (Applied Biosystems, Perkin Elmer, CA, USA). Sterile distilled water subjected to WGA was used a negative control and included in all plates. WGA amplified CpGenome™ Universal Methylated DNA (Merck Millipore, Burlington, MA, USA) subjected to six serial dilutions (5× factor dilution) was used to generate a standard curve in each plate, allowing for relative quantification and PCR efficiency evaluation. Efficiency values above 90% were considered. Relative methylation levels were determined as the ratio between the mean methylation levels of each gene and the respective value for *β-Actin* (the housekeeping gene), multiplied by 1000 for easier tabulation.

### 4.4. Statistical Analysis

Non-parametric tests were used to compare methylation levels of each gene promoter between cases and respective controls and to evaluate associations with clinicopathological features. Mann-Whitney U test was used for comparisons between two groups and Kruskall-Wallis test for three or more groups, followed by Mann-Whitney U test with Bonferroni’s correction for pairwise comparisons. Correlations between methylation levels and age were assessed by Spearman nonparametric correlation test. A *p* value < 0.05 was considered statistically significant.

For each gene, samples were categorized as methylated or unmethylated based on cut-off value determined using Youden’s J index (value combining highest sensitivity and specificity), through ROC curve analysis [56]. A positive result was considered when a sample was classified as methylated and negative when unmethylated. Validity estimates [sensitivity, specificity, positive predictive value (PPV), negative predictive value (NPV), and accuracy] were calculated to assess biomarker performance. Gene panels were constructed to maximize detection performance, considering a positive result whenever at least one gene promoter was methylated. The validity estimates for “PanCancer” panel were determined by assembling all cancer samples including BrC, CRC and LC (*n* = 253) vs. AC samples (*n* = 103). For “CancerType” panel, the cut-offs were determined by comparing each tumor type with the other two. A multiple ROC curves via resampling analysis was performed in order to calculate the validity estimates for “PanCancer” and “CancerType” panels, using a similar methodology previously described [57]. Briefly, the samples were randomly divided in a training (70%) and validation (30%) sets. The cut-off value comprising the highest sensitivity and specificity was estimated in the training set and the validity estimates were calculated in the validation set using that cut-off. This procedure was repeated 1000 times, and the mean of the sensitivities and specificities was calculated. These calculations were performed using R v3.4.4. Two-tailed *p*-values calculation and other ROC curve analyses were performed using a computer assisted program (SPSS Version 24.0, Chicago, IL, USA). Graphics were assembled with GraphPad 6 Prism (GraphPad Software, La Jolla, CA, USA).

## 5. Conclusions

A selected gene promoter methylation assessment in ccfDNA is shows promise for simultaneous screening of BrC, CRC and LC, the major causes of cancer-related morbidity and mortality in women. The panels might complement current screening modalities, perfect the triage of cancer suspects, and increase compliance and cost-effectiveness. Large-scale studies are now required to validate these findings and define the best algorithm for clinical application of these minimally-invasive methylation-based tests.

## Figures and Tables

**Table 1:** Clinicopathological features of BrC, CRC and LC patients and ACs enrolled in this study.

Clinicopathological Features	AC	Cancer Patients
Number	103	253
Age median (range)	52 (45–65)	63 (29–93)
		**Breast Cancer**
Histological Type	n.a.	
Invasive Carcinoma, no special type (NST)		80
Invasive lobular carcinoma		12
Ductal carcinoma in situ		7
Other invasive carcinoma subtypes ^a^		9
Primary Tumor (T)	n.a.	
Tis		7
T1&T2		95
T3&T4		6
Regional lymph node (N)	n.a.	
N0		65
N+		43
Distant metastasis (M)	n.a.	
M0		105
M1		3
Clinical Stage	n.a.	
0		7
I/II		88
III/IV		13
		**Colorectal Cancer**
Histological Type	n.a.	
Premalignant Lesions ^b^		3
Adenocarcinoma (all subtypes)		68
Neuroendocrine carcinoma		1
Tumor location		
Proximal colon	n.a.	23
Distal colon		30
Rectum		19
Primary tumor (T) ^c^	n.a.	
Tis		3
T1&T2		18
T3&T4		49
Regional lymph node (N) ^c^	n.a.	
N0		37
N+		33
Distant metastasis (M)	n.a.	
M0		66
M1		6
Clinical Stage	n.a.	
0		3
I/II		34
III/IV		35
		**Lung Cancer**
Histological Type	n.a.	
Non-small cell lung carcinoma (NSCLC)		
Adenocarcinoma		56
Other NSCLC subtypes ^d^		8
Small-cell lung carcinoma (SCLC)		8
Carcinoid tumor		1
Primary Tumor (T) ^e^	n.a.	
T1		18
T2/T3/T4		51
Regional lymph node (N) ^f^		
N0	n.a.	27
N+		45
Distant metastasis (M)	n.a.	
M0		36
M1		37
Clinical StageI/II	n.a.	
		21
III/IV		52

^a^ Includes medullary, mucinous and mixed type carcinoma (invasive carcinoma, NST and micropapillary carcinoma); ^b^ Includes tubulovillous adenoma with high-grade dysplasia and intramucosal adenocarcinoma; ^c^ No information available in 2 cases; ^d^ Includes squamous cell carcinoma and large-cell neuroendocrine carcinoma; ^e^ No information available in 4 cases; ^f^ Not possible to determine in 1 case; AC, Asymptomatic Control; n.a.: non-applicable

**Table 2:** Biomarker performance detection of “PanCancer” panel (*APC*, *FOXA1* and *RASSF1A*) in ccfDNA.

Validity Estimates	PanCancer
Sensitivity %	72.4
Specificity %	73.5
Positive Predictive Value %	87.1
Negative Predictive Value %	52.1
Accuracy %	72.8

**Table 3:** Methylated gene promoter combinations for BrC, CRC and LC discrimination using the “CancerType” panel.

Gene	BrC	CRC	LC
*SCGB3A1*	+	−	−
*SEPT9*	−	+	−
*SOX17*	−	−	+

“+” indicates a higher probability to find that cancer; “−” denotes that there is a low probability for that cancer type be present. Abbreviations: BrC—Breast Cancer; CRC—Colorectal Cancer; LC—Lung Cancer.

**Table 4:** Performance of gene promoter combinations for discrimination among BrC, CRC and LC.

Gene	Sensitivity %	Specificity %	Accuracy %
**BrC**			
*SCGB3A1*	16.8	80.0	53.0
*SEPT9*	-	-	-
*SOX17*	-	-	-
**CRC**			
*SCGB3A1*	-	-	-
*SEPT9*	11.1	98.9	73.9
*SOX17*	-	-	-
**LC**			
*SCGB3A1*	-	-	-
*SEPT9*	-	-	-
*SOX17*	39.4	85.1	71.9

Abbreviations: BrC—Breast Cancer; CRC—Colorectal Cancer; LC—Lung Cancer.

## Supplementary Materials

The following are available online at http://www.mdpi.com/2072-6694/10/10/357/s1, Table S1: Promoters’ methylation levels cut-off values used to categorize BrC, CRC and LC samples vs. AC samples used for validity estimates calculation in Figure 1 and Tables S2–S4. Table S2: Biomarker performance of each promoter’s gene methylation for BrC detection in ccfDNA, Table S3: Biomarker performance of each promoter’s gene methylation for CRC detection in ccfDNA. Table S4: Biomarker performance of each promoter’s gene methylation for LC detection in ccfDNA liquid biopsies. Table S5: Additional clinicopathological features of breast cancer (BrC) patients’ plasma samples used in this study, Table S6: Primers and probes sequences with respective fluorochrome and quencher.
