# Supplementary material for: Cell-Free DNA Methylation of Selected Genes Allows for Early Detection of the Major Cancers in Women
Source: Cancers (Basel). 2018 Sep 26;10(10):357. doi: 10.3390/cancers10100357 (PMC6210550; doi:10.3390/cancers10100357)
Supplement: Supplementary file 1 [file cancers-10-00357-s001.pdf]

# Supplementary Materials: Cell-free DNA Methylation of Selected Genes Allows for Early Detection of the Major Cancers in Women

**Sandra P. Nunes, Catarina Moreira-Barbosa, Sofia Salta, Susana Palma de Sousa, Inês Pousa, Júlio Oliveira, Marta Soares, Licínio Rego, Teresa Dias, Jéssica Rodrigues, Luís Antunes, Rui Henrique and Carmen Jerónimo**

**Table S1.** Promoters' methylation levels cut-off values used to categorize BrC, CRC and LC samples vs. AC samples used for validity estimates calculation in Figure 1 and Tables S2, S3 and S4.

| Genes | BrC vs. AC | CRC vs. AC | LC vs. AC |
|---|---|---|---|
| *APC* | 6.710 | 4.711 | 4.115 |
| *FOXA1* | 68.01 | 142.4 | 30.12 |
| *RARβ2* | 24.88 | 3.949 | 3.056 |
| *RASSF1A* | 58.27 | 39.47 | 26.57 |
| *SCGB3A1* | $4.130 \times 10^{-3}$ | $6.291 \times 10^{-5}$ | $6.140 \times 10^{-3}$ |
| *SEPT9* | 49.92 | 8.973 | 0 |
| *SOX17* | 184.7 | 0.6633 | 28.16 |

**Table S2.** Biomarker performance of each promoter's gene methylation for BrC detection in ccfDNA.

| Genes | Sensitivity % | Specificity % | PPV % | NPV % | Accuracy % |
|---|---|---|---|---|---|
| *APC* | 32·41 | 94·17 | 85·37 | 57·06 | 62·56 |
| *FOXA1* | 38·89 | 79·61 | 66·67 | 55·41 | 58·77 |
| *RASSF1A* | 19·44 | 100·0 | 100·0 | 54·21 | 58·77 |
| *SCGB3A1* | 21·30 | 92·23 | 74·19 | 52·78 | 55·92 |

Abbreviations: PPV—Positive Predictive Value; NPV—Negative Predictive Value.

**Table S3.** Biomarker performance of each promoter's gene methylation for CRC detection in ccfDNA.

| Genes | Sensitivity % | Specificity % | PPV% | NPV% | Accuracy % |
|---|---|---|---|---|---|
| *APC* | 20·83 | 94·17 | 71·43 | 62·99 | 64·00 |
| *FOXA1* | 50·00 | 88·35 | 75·00 | 71·65 | 72·57 |
| *RARβ2* | 16·67 | 95·15 | 70·59 | 62·03 | 62·86 |
| *RASSF1A* | 13·89 | 99·03 | 90·91 | 62·20 | 64·00 |
| *SCGB3A1* | 26·39 | 90·29 | 65·52 | 63·70 | 64·00 |
| *SEPT9* | 11·11 | 100·0 | 100·0 | 61·68 | 63·43 |
| *SOX17* | 23·61 | 90·29 | 62·96 | 62·84 | 62·86 |

Abbreviations: PPV—Positive Predictive Value; NPV—Negative Predictive Value.

**Table S4.** Biomarker performance of each promoter's gene methylation for LC detection in ccfDNA liquid biopsies.

| Genes | Sensitivity % | Specificity % | PPV% | NPV% | Accuracy % |
|---|---|---|---|---|---|
| *APC* | 35·62 | 94·17 | 81·25 | 67·36 | 69·89 |
| *FOXA1* | 72·60 | 73·79 | 66·25 | 79·17 | 73·30 |
| *RARβ2* | 24·66 | 95·15 | 78·26 | 64·05 | 65·91 |
| *RASSF1A* | 21·92 | 98·06 | 88·89 | 63·92 | 66·48 |
| *SOX17* | 38·36 | 95·15 | 84·85 | 68·53 | 71·59 |

Abbreviations: PPV—Positive Predictive Value; NPV—Negative Predictive Value.

**Table S5.** Additional clinicopathological features of breast cancer (BrC) patients' plasma samples used in this study.

| Clinicopathological features | BrC |
|---|---|
| Estrogen Receptor Status [a] | |
| Positive | 91 |
| Negative | 15 |
| Progesterone Receptor Status [b] | |
| Positive | 81 |
| Negative | 24 |
| ERBB2 Receptor Status [c] | |
| Positive | 13 |
| Negative | 84 |
| Molecular subtype [d] | |
| Luminal | 90 |
| ERBB2 overexpression (HR−, ERBB2+) | 4 |
| TNBC | 10 |

[a] Not amenable to obtain information for 2 cases; [b] No information available in 3 cases; [c] Not possible to determine in 11 cases; [d] No information available in 4 cases. Abbreviations: AC—Asymptomatic Control; ERBB2—Erb-B2 receptor tyrosine kinase; HR—Hormone Receptor; TNBC—Triple Negative Breast Cancer.

**Table S6.** Primers and probes sequences with respective fluorochrome and quencher.

| Gene | | Sequences |
|---|---|---|
| *ACTβ* | Primers | F – 5′ TGG TGA TGG AGG AGG TTT AGT AAG T 3′ |
| | | R – 5′ ACC AAT AAA ACC TAC TCC TCC CTT AA 3′ |
| | Probe | 5′ Cy5 – ACC ACC ACC CAA CAC ACA ATA ACA AAC ACA – QSY 3′ |
| *APC* | Primers | F – 5′ TGT GTT TTA TTG CGG AGT GC 3′ |
| | | R – 5′ CAC ATA TCG ATC ACG TAC GC 3′ |
| | Probe | 5′ VIC – CAATCGACGAACTCCCGAC – MGB 3′ |
| *FOXA1* | Primers | F – 5′ CGA CGT TAA GAC GTT TAA GC 3′ |
| | | R – 5′ CGC TCA ACG TAA ACA TCT TAC 3′ |
| | Probe | 5′ FAM-ATA TAC GAA TAA AAC GAC TTA ACG – MGB 3′ |
| *MGMT* | Primers | F – 5′ TTT CGA CGT TCG TAG GTT TTC GC 3′ |
| | | R – 5′ GCA CTC TTC CGA AAA CGA AAC G 3′ |
| | Probe | 5′ VIC – TGC GTA TCG TTT GCG – MGB 3′ |
| *RARβ2* | Primers | F – 5′ TCG AGA ACG CGA GCG ATT 3′ |
| | | R – 5′ GAC CAA TCC AAC CGA AAC 3′ |
| | Probe | 5′ HEX – CTT ACA AAA AAC CTT CCG AAT ACG TTC CGA – Iowa Black RQ-Sp 3′ |
| *RASSF1A* | Primers | F – 5′ AGC GAA GTA CGG GTT TAA TC 3′ |
| | | R – 5′ ACA CGC TCC AACC GA ATA 3′ |
| | Probe | 5′ NED – CGG GAG TTG GTA TTC GTT GGG CG – QSY3′ |
| *SCGB3A1* | Primers | F – 5′ GTA CGG TCG TGA GCG GAG C 3′ |
| | | R – 5′ GAA ACT TCT TAT ACC CGA TCC TC 3′ |
| | Probe | 5′ FAM – GCC GAC CTC GCC CGC GCT CCT AAA – Iowa Black RQ-Sp 3′ |
| *SEPT9* | Primers | F – 5′ TTA GTT AGC GCG TAG GGT TC 3′ |
| | | R – 5′ ACC TTC GAA ATC CGA AAT AA 3′ |
| | Probe | 5′ NED – GCG TTA ACC GCG AAA TCC GAC ATA ATA ACT – QSY 3′ |
| *SHOX2* | Primers | F – 5′ ATT CGT ATT TGG TCG CGT AC 3′ |
| | | R – 5′ CTA CTA CGA CCG CCA CTA CC 3′ |
| | Probe | 5′ FAM – CAA CGT AAC GAA CG – MGB 3′ |
| *SOX17* | Primers | F – 5′ GAT CGG TTC GTT TTC GTC G 3′ |
| | | R – 5′ GCC CGT ATT CTA ACC TAT CG 3′ |
| | Probe | 5′ Cy5 – ACC GAC CTA ATA ACA CTA CGA ACG C – Iowa Black RQ-Sp 3′ |

© 2018 by the authors. Licensee MDPI, Basel, Switzerland. This article is an open access article distributed under the terms and conditions of the Creative Commons Attribution (CC BY) license (http://creativecommons.org/licenses/by/4.0/).
